# Validity and reliability of the tuberculosis-related stigma scale version for Brazilian Portuguese

**DOI:** 10.1186/s12879-017-2615-2

**Published:** 2017-07-21

**Authors:** Juliane de Almeida Crispim, Laís Mara Caetano da Silva, Mellina Yamamura, Marcela Paschoal Popolin, Antônio Carlos Vieira Ramos, Luiz Henrique Arroyo, Ana Angélica Rêgo de Queiroz, Aylana de Souza Belchior, Danielle Talita dos Santos, Flávia Meneguetti Pieri, Ludmila Barbosa Bandeira Rodrigues, Simone Terezinha Protti, Ione Carvalho Pinto, Pedro Fredemir Palha, Ricardo Alexandre Arcêncio

**Affiliations:** 1 Maternal-Infant and Public Health Nursing Department, College of Nursing of Ribeirão Preto, University of São Paulo, Av dos Bandeirantes 3900, Vila Monte Alegre 14040-902 Ribeirão Preto, São Paulo, Brazil; 2 Department of Nursing, Federal University of Santa Maria, Santa Maria, Rio Grande do Sul Brazil; 3 Department of Nursing, Londrina State University, Londrina, Paraná, Brazil; 4 Institute for Health Sciences, Federal University of Mato Grosso, Sinop, Mato Grosso, Brazil; 5 Department of Nursing, Federal University of São Carlos, São Carlos, São Paulo, Brazil

**Keywords:** Social stigma, Validation studies, Tuberculosis

## Abstract

**Background:**

Stigma associated with tuberculosis (TB) has been an object of interest in several regions of the world. The behaviour presented by patients as a result of social discrimination has contributed to delays in diagnosis and the abandonment of treatment, leading to an increase in the cases of TB and drug resistance. The identification of populations affected by stigma and its measurement can be assessed with the use of valid and reliable instruments developed or adapted to the target culture. This aim of this study was to analyse the initial psychometric properties of the Tuberculosis-Related Stigma scale in Brazil, for TB patients.

**Methods:**

The Tuberculosis-Related Stigma scale is a specific scale for measuring stigma associated with TB, originally validated in Thailand. It presents two dimensions to be assessed, namely Community perspectives toward tuberculosis and Patient perspectives toward tuberculosis. The first has 11 items regarding the behaviour of the community in relation to TB, and the second is made up of 12 items related to feelings such as fear, guilt and sorrow in coping with the disease. A pilot test was conducted with 83 TB patients, in order to obtain the initial psychometric properties of the scale in the Brazilian Portuguese version, enabling simulation of the field study.

**Results:**

As regards its psychometric properties, the scale presented acceptable internal consistency for its dimensions, with values ≥0.70, the absence of floor and ceiling effects, which is favourable for the property of scale responsiveness, satisfactory converging validity for both dimensions, with values over 0.30 for initial studies, and diverging validity, with adjustment values different from 100%.

**Conclusion:**

The results found show that the Tuberculosis-Related Stigma scale can be a valid and reliable instrument for the Brazilian context.

## Background

Tuberculosis-related stigma has been studied around the world. Patients’ behaviour in response to social discrimination has contributed to diagnostic delays and the abandonment of treatment, resulting in an increased number of multidrug-resistant tuberculosis (TB) cases [1, 2].

Among the political and social commitments to reduce the stigma, the identification of affected populations is highlighted, as well as the assessment of the stigma by means of valid and reliable tools, developed or adapted for the target culture. The use of these instruments in different countries and languages contributes to the identification of the health needs, decision taking and care improvements, besides enhancing the communication among researchers and professionals from different cultures [3].

Few instruments were found in the literature that serve to measure tuberculosis-related stigma and were subject to validity and reliability assessments [4–6]. No formally adapted scales exist in Brazil for measuring TB-related stigma.

To analyse the determining factors of this stigma, identify the intervention points and assess the quality of care and its effectiveness in the reduction of the stigma, Van Rie et al. developed a scale called the Tuberculosis-Related Stigma scale, which measures tuberculosis stigma in quantitative terms [6].

The Tuberculosis-Related Stigma scale was built in southern Thailand and its initial items were selected based on the following key domains: fear of transmitting the disease, values and attitudes associated with shame, guilt, judgment and revelation of one’s status, obtained through the literature review. After interviews and focus groups with patients, relatives, health professionals and community members, the scale was adapted culturally and linguistically for use in Brazil [6].

The scale was translated into English, Thai and Malayan (Austronesian language of the Malay people and those of other ethnic origins living in southern Thailand) with satisfactory internal consistency (Cronbach’s alpha >0.70). The construct validity showed an inverse correlation with social support, confirming the statistical hypothesis the authors presented in the validation process.

In view of the disease’s current epidemiological reality in the country and around the world, in which the sociocultural aspects involved in the early diagnosis and cure of TB are increasingly important, and as there are no tools available in Brazilian Portuguese to measure it, the validation of the Tuberculosis-Related Stigma scale for use in Brazil is both appropriate and relevant. The objective in this study was to analyse the initial psychometric properties of the Tuberculosis-Related Stigma scale in Brazil for TB patients.

## Methods

### Study design and place of study

A methodological study with cross-sectional design was undertaken in an endemic Brazilian city. Located in the north-east of the state of São Paulo, 313 km from the state capital and 706 km from Brasília, the city has an estimated population of 658,059 inhabitants within a territory of 650.92 km^2^ (IBGE, 2014). With a Human Development Index (HDI) of 0.80 and an illiteracy rate of 3.0, according to the São Paulo Social Vulnerability Index (IPVS), the city fits into the group with good social and economic indicators [7, 8].

In the city, TB patient care is concentrated in the referral outpatient clinics that offer a TB control program, distributed across the five health districts (East, West, North, South and Central). These services operate with a specialized team, consisting of at least one physician, two auxiliary nurses and one baccalaureate nurse. Their activities are focused on the diagnosis, clinical management of cases and contacts, medical appointment for control and Directly Observed Treatment (DOT).

As regards the epidemiological situation of TB in the year before the data collection in this study (2013), the city presented an incidence rate of 28.2 cases per 100,000 inhabitants, a mortality rate of 1.8 deaths per 100,000 inhabitants and a cure rate of 77.8%, below the target set by the WHO by 85% [9, 10].

### Population and sample

The reference population for this study included TB patients monitored at the city’s referral outpatient clinics. As far as the sample design for the study phases is concerned, the authors [11] consider 50 observations to be sufficient for the research proposal. The final sample consisted of 83 subjects, successively selected during home visits.

The following inclusion criteria were adopted: TB patients aged 18 years or older, under treatment at the referral outpatient clinics of the city for at least two weeks. This period was chosen because it corresponds to the team’s adaption to the treatment, allowing the researchers to articulate between the health service and the patients, obtaining their agreement to participate in the study.

Patients without the minimal skills required to understand the scale questions and patients who did not feel comfortable talking about the research subject were excluded.

### Scale

The Tuberculosis-Related Stigma scale is a specific measure of the stigma related to TB applied to patients. It consists of two dimensions, known as Community perspectives on TB and Patient perspectives on TB. The Community perspectives regarding tuberculosis include 11 items on the community’s behaviour towards TB patients, and the Patient perspectives toward tuberculosis consist of 12 items related to feelings such as fear, guilt and sorrow in coping with the disease.

The scale consists of items written affirmatively in Brazilian, graded on a four-point Likert scale (1 to 4), ranging from I strongly disagree (1) to I disagree (2), I agree (3) and I strongly agree (4). The item scores from the questionnaire were summed and the scores ranged respectively from 11 to 44 for Community perspectives on tuberculosis to 12–48 for Patient perspectives on tuberculosis, with 11 indicating no stigma and both 44 and 48, depending on the dimension, indicating the highest level of TB stigma [6].

### Translation of the scale

Two translators who were fluent in the English language and were native speakers in Portuguese translated the scale independently. After that, the translated versions were analysed in terms of agreement, and the disagreement items were evaluated by the research group and adjusted. Back translation into the original language was then carried out by two native speakers of the English language who did not know the objectives of the study. The consensus version in English was subsequently compared with the original version of the scale and analysed/approved by the project leader in southern Thailand [12].

### Pilot test

The pilot test was intended to collect data on the initial psychometric properties of the scale in the Brazilian Portuguese version and permitted a simulation of the field study [13]. Therefore, the scale version adapted to Brazil was applied to a sample of 83 TB patients under treatment at the four referral outpatient clinics of the city where the study was undertaken.

The data were collected between April 2015 and April 2016. As in the semantic validation phase, researchers affiliated with the project collected the data after background training by the lead researcher during meetings of the research group.

Double-data entry was used to avoid possible transcription errors. The software adopted was the Statistical Package for the Social Sciences (SPSS), version 19.0. In the construct validity, in addition, the Multitrait Analysis Program (MAP) was adopted [14].

The presence of the floor and ceiling effects was verified, bearing in mind that acceptable information on these effects can be observed in a sample consisting of at least 50 subjects [15]. When they are present (15% of the answers are concentrated in the lowest or highest scale score), this can negatively affect the responsiveness of the instrument [16].

To analyse the construct validity, the convergent and divergent validity of the scale were used, by means of multitrait-multimethod analysis, with the aim of verifying the correlations between items and dimensions through MAP. According to Fayers and Machin (2007) [17], acceptable correlation coefficients for convergent validity correspond to 0.30 for initial studies and are above 0.40 for final studies [15]. To analyse the divergent validity by means of MAP, it was verified how many times (expressed in percentage form) the correlation between an item and the dimension it belongs to is statistically higher than its correlation with the dimension it does not belong to.

The reliability of the scale was measured using Cronbach’s alpha. The coefficients were estimated, considering results above 0.70 as acceptable [15, 18].

### Ethical aspects

Approval for the study was obtained from the Ethics Committee at the University of São Paulo at Ribeirão Preto College of Nursing under CAAE 24841413.5.0000.5393. All participants signed the Free and Informed Consent Form.

## Results

Eighty-three patients who were under treatment for TB at the four referral outpatient clinics participated in the study. The minimal sample at each service consisted of seven patients and one patient refused to participate. As regards the patients’ sex, 56 (67.5%) were men and 27 (32.5%) women. As far as professional occupation is concerned, 38 (45.8%) were unemployed at the time of the data collection. The age ranged between 19 and 92 years, with a mean age of 43 (SD 13.9); the mean length of education was about seven years and the mean family income was less than two minimum wages per month.

In terms of the clinical variables, ten patients were HIV-positive and 71 (85.5%) suffered from pulmonary TB, the majority being new cases without previous treatment, as shown in Table 1.

**Table 1:** Sociodemographic characteristics and clinical variables of participants (*n* = 83)

Sociodemographic	*n* (%)	
Characteristics		
Sex		
Female	27 (32.5)	
Male	56 (67.5)	
Origin		
Northeast	8 (9.6)	
Central-West	1 (1.2)	
Southeast	72 (86.8)	
South	2 (2.4)	
Marital Status		
Married	29 (34.9)	
Single	33 (39.8)	
Widowed	6 (7.2)	
Divorced	6 (7.2)	
Other	9 (10.9)	
Ethnic origin		
White	43 (51.8)	
Black	19 (22.9)	
Mulatto	21 (25.3)	
Occupation		
Registered employee	9 (10.8)	
Self-employed	17 (20.5)	
Retired	7 (8.4)	
Unemployed	38 (45.8)	
Other	12 (14.5)	
	$\overline{\mathrm{x}}$	SD
Age (years)	42,7	13.9
Years of Study	6,8	3.3
Income (^*^)	1328,3	823.9
Clinical Variables	*n* (%)	
HIV		
Negative	73 (87.9)	
Positive	10 (12.1)	
Type of TB		
Pulmonary	71 (85.5)	
Extrapulmonary	12 (14.5)	
Case situation		
New case without previous treatment	75 (90.4)	
Relapse after cure	6 (7.2)	
Return after treatment abandonment	2 (2.4)	

(^*^) Minimum wage valid at the time of data collection: R$ 788.00 (2015) and R$ 880,00 (2016)

To calculate the scores, the syntax proposed by the DISABKIDS® group was used [19]. In Table 2, the means, medians, standard deviation, minima and maxima of the Tuberculosis-Related Stigma scale scores are displayed. As observed, the mean scores in both dimensions are higher than 50%.

**Table 2:** Means, medians, standard deviation, minima and maxima of the Tuberculosis-Related Stigma scale scores

	Tuberculosis patients					
Dimensions	Mean	%	Median	SD	Minimum	Maximum
Community perspectives on tuberculosis	2.6	83.1	2.5	0.2	2.3	3.3
Patient perspectives on tuberculosis	2.6	62.7	2.6	0.2	2.1	2.9

As for the floor and ceiling effects, in Table 3, the absence of these effects in the dimensions is observed; that is, no answer concentrations higher than 15% were found at the top and bottom of the scale, which favours its responsiveness.

**Table 3:** Analysis of floor and ceiling effects of answers in Tuberculosis-Related Stigma dimensions

	Tuberculosis patients	
Dimensions	% answers in minimum score	% answers in maximum score
Community perspectives on tuberculosis	1.0	1.4
Patient perspectives on tuberculosis	0	1.3

The reliability was analysed according to the internal consistency, determined using the Cronbach’s alpha coefficient. The coefficients if excluded the item and the total alpha of the scale dimensions are displayed in Table 4.

**Table 4:** Alpha coefficients if excluding the item and total Alpha of the dimensions

Community perspectives on tuberculosis (Item)	Cronbach’s Alpha if excluding the item
1. Some people prefer not to have individuals with TB living in their community	0.72
2. Some people keep distance from TB patients	0.71
3. Some people think people with TB are disgusting	0.69
4. Some people feel comfortable when they are close to a person with TB^a^	0.71
5. Some people do not want people with TB playing with their children	0.66
6. Some people want to talk with those who have TB^a^	0.65
7. If a person has TB, some members of the community will behave differently in relation to that person for the rest of their lives	0.68
8. Some people may not want to eat or drink with friends that have TB	0.66
9. Some people do not avoid touching people with TB^a^	0.69
10. Some people may not want to eat or drink with family members who have TB	0.62
11. Some people do not fear those who have TB^a^	0.67
Total alpha	0.70
Patient perspectives on tuberculosis (Item)	
1. Some people with TB feel guilty because their family carries the burden of taking care of them	0.68
2. Some people with TB keep distance from other people in order to avoid the transmission of TB germs	0.73
3. Some people that have TB do not feel lonely^a^	0.70
4. Some people with TB feel hurt with the way other people react when they find out that they have TB	0.67
5. Some people with TB lose friends when they share the information that they have the disease	0.68
6. Some people with TB are not worried about the possibility of having Aids too^a^	0.74
7. Some people with TB fear telling people out of their families that they have the disease	0.65
8. Some people with TB will carefully choose those who they will inform about their condition	0.66
9. Some people with TB do not fear going to TB clinics because other people may see them there^a^	0.73
10. Some people with TB do not fear telling their families that they have the disease^a^	0.69
11. Some people with TB fear telling other people about their condition because other people may think they have Aids too	0.67
12. Some people do not feel guilty as they may have been affected by TB due to the habit of smoking, drinking alcohol or not taking care of themselves^a^	0.70
Total alpha	0.71

^a^Items written in inverse form

When testing the impact of removing each item on the total alpha coefficient of the scale dimensions, changes were observed, ranging from 0.62 (Some people may not want to eat or drink with family members who have TB) to 0.72 (Some people prefer not to have individuals with TB living in their community) in the first dimension, and from 0.65 (Some people with TB fear telling people outside their families that they have the disease) to 0.74 (Some people with TB are not worried about the possibility of having Aids too) in the second dimension.

The Cronbach’s alpha was considered good for both the dimension Community perspectives on TB (0.70) and the dimension Patient perspectives on TB (0.71).

With regard to the construct validity, verified using the convergent and divergent validity of the scale, Pearson’s correlation coefficients were used between the items and each of their dimensions. Statistical significance for the correlation coefficients in the validity was verified using Multitrait-multimethod analysis. The convergent validity coefficients in each dimension are displayed in Table 5.

**Table 5:** Pearson’s Correlation Coefficients between items and each dimension of the Tuberculosis-Related Stigma scale

	TB patients	
Item	Community perspectives on tuberculosis	Patient perspectives on tuberculosis
01	0.09	0.26
02	0.14	- 0.04
03	0.28	0.05
04	0.14	0.42
05	0.44	0.18
06	0.55	0.48
07	0.33	0.07
08	0.47	0.03
09	0.27	0.19
10	0.71	0.19
11	0.44	0.18
01	0.38	0.45
02	0.11	0.01
03	0.20	0.32
04	0.33	0.48
05	0.43	0.46
06	- 0.18	0.03
07	0.26	0.62
08	0.18	0.60
09	- 0.09	0.02
10	0.16	0.40
11	0.38	0.49
12	- 0.16	0.27

The analysis of the convergent validity coefficients (Table 6) reveals that most items in the first dimension satisfactorily complied with the criterion adopted (> 0.30 for initial studies), except for items 01, 02 and 04. As regards the items in the second dimension, items 02, 06 and 09 obtained coefficients <0.30, but the remaining items reached acceptable correlation coefficients for convergent validity.

**Table 6:** MAP analysis results for divergent validity of Tuberculosis-Related Stigma scale

	Tuberculosis patients				
Dimensions	-2^a^ (n items /%)	-1^b^ (n items /%)	1^c^ (n items /%)	2^d^ (n items/%)	Adjustment (%)
Community perspectives on tuberculosis	1 (9.1)	1 (9.1)	3 (27.3)	6 (54.5)	81.8
Patient perspectives on tuberculosis	0 (0)	4 (33.3)	5 (41.7)	3 (25.0)	66.7

^a^Correlation between item and dimension it belongs to is significantly lower than its correlation with the dimension it does not belong to;

^b^Correlation between item and dimension it belongs to is lower than its correlation with the dimension it does not belong to;

^c^Correlation between item and dimension it belongs to is higher than its correlation with the dimension it does not belong to;

^d^Correlation between item and dimension it belongs to is significantly higher than its correlation with the dimension it does not belong to

For the divergent validity, the results of both scale dimensions presented coefficients different from 100% (Table 6).

## Discussion

The objective of the study was to test the initial psychometric properties of the version of the Tuberculosis-related stigma scale adapted to Brazilian Portuguese, with a view to offering an instrument to measure and assess the TB-related stigma. Therefore, the guidelines found in international and Brazilian literature were followed [19–21].

The psychometric properties obtained through the pilot test, which included a sample of 83 patients under treatment for TB at the four referral outpatient clinics, permitted a preliminary analysis of the reliability and validity of the scale version adapted for use in Brazil.

With respect to the descriptive statistical measures of the scale, the mean score in each dimension was higher than the mean score for the entire scale, with 83.1% for the dimension Community perspectives on TB and 62.7% for the dimension Patient perspectives on TB.

With regard to the presence of floor and ceiling effects, in this study, these were not found in any of the scale dimensions. This predicts good responsiveness [17], an important characteristic in detecting changes in TB patients’ health condition over time, related to the presence or absence of stigma.

Authors [22] consider that the approach based on statistical distribution, i.e. measures based on the longitudinal distribution of the sample, is one of the measures for assessing the responsiveness. Any change in the variability of the scores, i.e. floor and ceiling effects, can minimize the responsiveness to changes.

Although there is no clarity about the type of change a responsible scale should be capable of detecting, i.e. changes related to treatment compliance and continuity or changes in the current value of the construct studied, psychosocial measures such as stigma can be used in the clinical assessment of TB patients. Nevertheless, it is essential to demonstrate its accurate detection of changes over time, by means of valid and reliable tools.

As regards the reliability of the scale, these findings demonstrated that the Tuberculosis-Related Stigma scale presents good coefficients in both dimensions (0.70 and 0.71). In the version used in southern Thailand, the coefficient corresponds to 0.88 in the Community dimension and 0.82 in the Patient dimension. The coefficients in Brazil are lower than those found in southern Thailand, but indicate the internal consistency of the scale according to criteria proposed in the literature, with scores ranging between 0.7 and 0.95, therefore the scale is really appropriate to the population of the study [15].

It is important to highlight that an excellent internal consistency in scales with more than one dimension (multidimensionality) indicates that the items in the different dimensions of a scale are strongly correlated, although the relation between the dimensions is inferior to that observed between the component items [18].

Cronbach’s alpha is a useful indicator for investigating the reliability of a measure, and thus permits the precision of an instrument to be studied. Nevertheless, this estimate is subject to different influences that should be interpreted with caution [18].

According to authors [23], when calculating Cronbach’s alpha, each item should be tested individually in relation to the other scale items. Thus, an item should be eliminated from the scale if the final alpha coefficient of the scale is higher without it. In a strictly psychometric analysis of the impact that the removal of each item would have on the total alpha of the dimension Patient perspectives toward tuberculosis, it was observed that items 6 and 9, written inversely, possess low item-total coefficients and should be eliminated. Both items translate behaviours that represent the TB-associated stigma, though, and keeping them in the scale does not represent any great loss of internal consistency, which can be tested in larger samples to verify whether these preliminary findings are coherent.

One author changed the order of the question, using positive and negative questions in the same instrument, and detected that this procedure confounds the respondents, advising against its use [24]. When inverting the order of the question, the items may not be perceived in exactly the opposite sense, which results in a lower reliability and validity of the data [25].

To study the construct validity, convergent and divergent validity were used, in which a linear correlation exists between the items of the scale version adapted to Brazil, mostly above 0.30, and the dimension they belong to (convergent validity), which is ideal for initial studies [17].

In the divergent validity, the results of both dimensions presented coefficients different to 100%. According to Fayers and Machin (2002) [17], the closer to 100%, the better the divergent validity. We believe that this kind of data can be related to the following aspects: a) subjectivity of the measure; b) background experience with internalized stigma. These aspects could be considered in future studies for analysing these results.

According to Link and Phelan (2001) [26], the distinct coefficients related to the stigmatization process, the different operations of these concepts, and the lack of particularity of the measures and constructs used make it impossible to construct syntheses that evidence how stigma influences people’s lives.

Nevertheless, the use of scales to assess the social stigma related to TB allows an explanation of why this can be a predictive factor of diagnostic delays and non-compliance with treatment in some contexts and not in others. This serves as a tool for assessing and redirecting resources to strengthen social support networks that involve intersectoral TB actions in health services [27].

In addition, the scale might be used as technology in TB care as algorithms screening for TB diagnosis or detection [28]. In this case, it will be able to be used to stratify the risk of TB stigma and support health-care professionals and health managers in taking decisions about more appropriate interventions to face the prejudices and myths related to TB as well to raise the awareness of the patients, their families and communities that the disease has a cure since the patients have a prompt diagnosis, social support and worthwhile treatment. Although the negative effect of stigma in the disease course is recognized, unfortunately there are still not enough tools to help health-care professionals manage the problem.

Brazil has a low treatment success rate of 71% even though it has adopted the DOTS as a universal strategy for all TB patients, and the stigma is probably an explanation for that. The WHO has launched an End TB strategy in which, among three pillars, one requires social protection as a resource for alleviating the situation of TB patients in terms of poverty and catastrophic expenditures [29].

However, social protection is more than just cash and social transfers, it includes the transformation of the social environment in which people live and also tackling stigma and discrimination just as has been done with HIV [30]. Therefore the study can contribute to moving TB care out of the box [31].

Among the study limitations, it is important to consider the small sample size, which is why confirmatory factor analysis could not be applied; it requires at least 10 subjects for each scale item [32]. Another limitation is that Brazil is a huge country, with cultural, historical and social differences, which might influence the cultural adaptation and validation of the Tuberculosis-Related Stigma scale.

Therefore, a methodological study was proposed to serve as a reference for Brazilian researchers for the mapping and monitoring of social stigma in different Brazilian regions. The use of specific tools can entail advances for care by taking into account psychosocial aspects, beyond the patient’s own expectations concerning his disease.

## Conclusion

The scale presented favourable initial psychometric properties in the pilot test, which sustains the validity and reliability of the scale in the Brazilian context. It would be interesting to apply the scale more widely to confirm through more robust statistical tests the relevance of the scale for Brazil. It was the first scale validated in the Portuguese language, which might be helpful for other Portuguese-speaking countries that are equally affected by tuberculosis and stigma as the African countries since the scale is adapted and assessed for their contexts. In any case, the Tuberculosis-Related Stigma scale can stimulate the advancement of operational research and the development of strategic actions to reduce the stigma of TB.
